# Louis Pasteur continues to shape the future of microbiology

**DOI:** 10.1242/dmm.050011

**Published:** 2022-12-12

**Authors:** Serge Mostowy

**Affiliations:** Department of Infection Biology, London School of Hygiene and Tropical Medicine, London, WC1E 7HT, UK

**Keywords:** Antimicrobial resistance, COVID-19, Microbiology

## Abstract

Louis Pasteur made seminal discoveries in microbiology, immunology and vaccinology that transformed clinical science and saved millions of lives. Since the 19th century, our ability to study infectious disease has undergone radical changes due to newly emerging technologies and infection models. In this Editorial, I consider Pasteur's impact on our ability to understand and combat infectious disease in the context of two modern-day pandemics: coronavirus disease 2019 (COVID-19) and antimicrobial resistance (AMR). During the COVID-19 pandemic, we witnessed remarkable ambition to understand severe acute respiratory syndrome coronavirus 2 (SARS-CoV-2) infection and to innovate effective vaccines to prevent disease. For the comparatively overlooked pandemic of AMR, we require the same level of urgency to develop alternative approaches to combat antibiotic-resistant bacterial strains that cause millions of deaths annually. Pasteur's statement “chance only favours the mind which is prepared” is a principle that captures ‘l'esprit Pasteur’. This principle should continue to guide modern-day research on infectious disease, and for this we need to support the development of predictive disease models and cutting-edge mechanistic research that prepare us for discovery and therapeutic impact.

## Teaser

**Summary:** The 200th anniversary of the birth of Louis Pasteur is an opportunity to revisit the origins of microbiology and to consider Pasteur's impact on our ability to combat infectious disease.

## The Story of Louis Pasteur

**Fig. 1.**
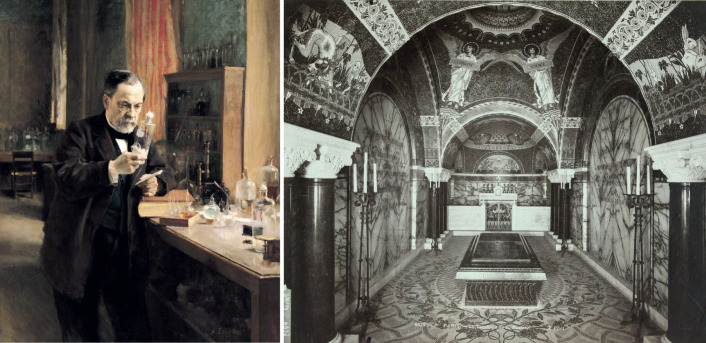
**Louis Pasteur, the visionary.** (Left) Louis Pasteur in his laboratory, 1885 (oil on canvas). Image courtesy of the Institut Pasteur. Image reproduced under the terms of the Attribution-NonCommercial-NoDerivs 2.0 Generic (CC BY-NC-ND 2.0) license. (Right) The tomb erected in memory of Louis Pasteur at the Institut Pasteur. Image courtesy of Petit Palais. Image reproduced under the terms of the Public Domain Dedication (CC0 1.0).

Louis Pasteur (1822-1895) was a French chemist interested in the origins of life (Kolter, 2021) (Fig. 1, left). During his career he made several seminal discoveries, enabling the process of microbial fermentation and the invention of pasteurisation (Manchester, 1995). He established causative relationships between microbes, infection and disease, leading to the ‘germ theory of disease’, which revolutionised clinical science (Casanova and Abel, 2013). Furthermore, Pasteur pioneered the use of attenuated microbes for vaccination, and saved millions of lives through the development of vaccines for anthrax and rabies (Barranco, 2020). By discovering principles of microbial fermentation and pasteurisation, germ theory and vaccination, Pasteur fundamentally changed how we view and combat infectious disease.

**Fig. 2.**
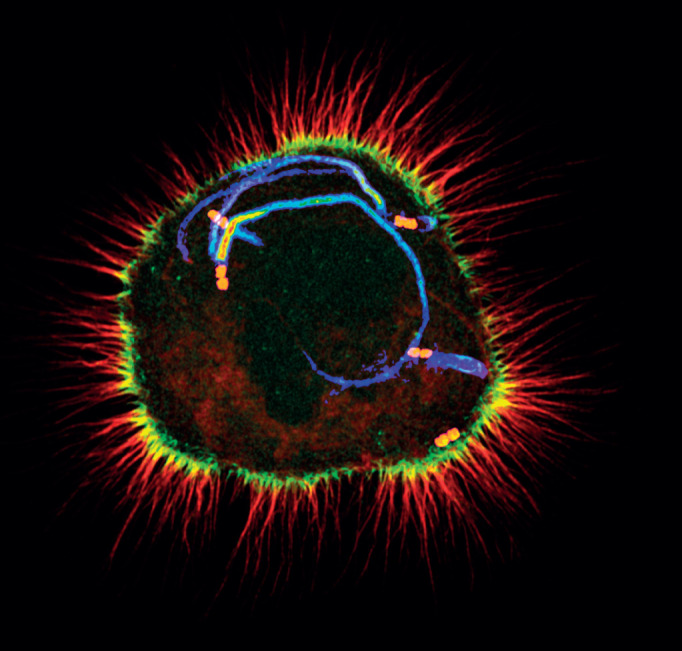
**Airyscan confocal image of a HeLa cell infected with *Shigella flexneri*, which are forming actin tails.** Bacterial DNA is shown in orange, SEPT7 in green and F-actin in red. *Shigella* MreB helps to position IcsA to form actin tails (highlighted with a ‘fire’ look-up table to reflect signal intensity). Image taken by Ana Teresa López Jiménez, reproduced from the cover of J. Cell Sci. (2019) 132(9). See article by Krokowski et al. (2019).

Pasteur's discoveries and contribution to medicine place him among the microbiology elite (Ligon, 2002). His life was made into a fictional film, ‘The Story of Louis Pasteur’ (1936), an Oscar award-winning account of his ability to conquer deadly diseases. Despite his unique understanding of the infection process, two of Pasteur's five children died from typhoid fever aged 9 and 12, highlighting that infectious disease was a major source of childhood mortality at the time. Building on the success of his vaccines, Pasteur was founder and director of the first Institut Pasteur (Paris), established in 1887 and internationally recognised for cutting-edge biomedical and translational research. Pasteur's remains are buried in a vault beneath the institute (Fig. 1, right), where I worked on cytoskeleton–bacteria interactions as a postdoctoral researcher in the Bacteria-Cell Interactions Unit (Fig. 2), headed by pioneering cellular microbiologist Pascale Cossart (2022).

Considering the many advances in technology, including complete genome sequencing and high-resolution microscopy techniques, alongside innovation in infection models, such as organoids and transgenic zebrafish, the field of microbiology is blooming more than ever. Here, I focus on two modern-day pandemics – coronavirus disease 2019 (COVID-19) and antimicrobial resistance (AMR), which are having a dramatic impact on science and society – to illustrate how Pasteur's legacy can potentiate breakthroughs in clinical medicine.

## Preparing for pandemics beyond COVID-19

Over the past ∼3 years, severe acute respiratory syndrome coronavirus 2 (SARS-CoV-2) has infected hundreds of millions of people, with deaths in ∼1% of those people [see World Health Organization (WHO) COVID-19 dashboard]. During this time, there have been remarkable advances in our understanding of the pathogenesis of the resultant disease, COVID-19, and swift development of vaccines with proven efficacy in preventing transmission and severe symptoms of the disease. To deliver such extraordinary feats, researchers needed predictive experimental systems, including *in vitro* and *in vivo* models, to fast track infection biology studies (Hooper and Patton, 2021; Jeong et al., 2022; Leist et al., 2020; van der Vaart et al., 2021).

It is valuable to consider the founding principles of vaccinology, established by Pasteur (Barranco, 2020), that enabled us to combat this infection. Pasteur's early vaccines inspired the development of a plethora of vaccines that, for example, have eradicated smallpox and (almost) eradicated polio and measles. Despite this progress, cases of vaccine-preventable diseases, such as measles, continue to rise in some parts of the world (see WHO data). In this way, it is paramount for society to learn from the past and make every effort to minimise COVID-19 by maximising vaccine uptake and global distribution.

The public demand for rapid solutions in response to COVID-19 also highlights our need to progress fundamental research that can be more immediately translated into human health impact. To enable this, we need to support researchers using innovative infection models for discovery and prevention of emerging, neglected and potentially pandemic infectious diseases, including West Nile, dengue and Zika virus diseases arising from vector-borne pathogens. In the future, we must exploit common and distinctive features from a wide variety of exotic diseases and host immune responses. This paradigm shift could illuminate new possibilities for combatting the COVID-19 pandemic and promoting our ability to control future emerging threats.

## Combatting AMR

AMR represents another ongoing pandemic, with successful therapy for human bacterial infections being challenged by antibiotic-resistant bacterial strains that cause millions of deaths annually (see WHO information). For example, in 2019, ∼5 million people died from AMR-related illnesses, and, of these, ∼1.3 million deaths were the direct result of AMR (Murray et al., 2022). To address this, we should be funding research initiatives to develop new strategies and promoting government policies to prevent the inappropriate use of antibiotics. The PASTEUR act is a bill that many charitable foundations are hoping will be passed in the United States, as it aims to incentivise companies to invest more in alternative approaches to fight AMR. In line with this, there are other initiatives in the United Kingdom and Europe, including The Fleming Fund and Disseminating Innovative Solutions for Antibiotic Resistance Management (DISARM). However, despite increasing public attention and funding initiatives, the subject of AMR has not yet earned a similar amount of attention as the COVID-19 pandemic. In some cases, not only is the problem of AMR being neglected, but government policies, such as the UK government's proposed plan for antibiotic prescription without a consultation with a GP, can threaten to exacerbate the issue (see Microbial Society response).

As observed from the response to the COVID-19 pandemic, solving the comparatively overlooked pandemic of AMR will also require an international effort and creative strategies (Joshi, 2022). As one example, bacteriophages, which are viruses that infect bacteria and replicate lytically, are being engineered and used therapeutically to combat infections with antibiotic-resistant bacterial strains (Cook and Wright, 2022). Bacteriophages were discovered in 1917 by Félix d'Hérelle, a French-Canadian microbiologist who was strongly influenced by Pasteur and was working internationally through the Institut Pasteur (1911-1921). d'Hérelle first experimented with phage therapy, which was, at the time, a pioneering example of applied microbiology (Summers, 2016). Since then, the study of bacteriophage–bacteria interactions has led to revolutionary advances, including the development of CRISPR gene-editing technology for which Emmanuelle Charpentier and Jennifer Doudna were awarded the Nobel Prize in Chemistry (2020). In this monumental breakthrough, Pasteur's legacy persists, as Charpentier was a graduate student (1992-1995) and postdoctoral researcher (1995-1996) at the Institut Pasteur, investigating molecular mechanisms underlying AMR. Now, it is exciting to witness the increasing number of successful phage therapy trials being performed in humans, as well as the mechanistic understanding being illuminated from investigation in animal disease models. As one example, a young cystic fibrosis (CF) patient chronically infected with a multidrug-resistant mycobacterial strain was administered genetically engineered mycobacteriophages, which resulted in clinical improvement (Dedrick et al., 2019). To further elucidate the mechanism of this treatment, a paper in Disease Models & Mechanisms (DMM) tested the mycobacteriophage and antibiotic combination therapy in a zebrafish CF model, also demonstrating enhanced mycobacterial clearance and establishing the zebrafish model as an ideal tool to investigate novel phage therapies (Johansen et al., 2021).

The study of bacteriophage–bacteria interactions has also led to the principle that innate immune mechanisms of human cells have bacterial origins (Wein and Sorek, 2022). Thus, it is of great interest to continue to explore common defence principles in bacteria and humans, and ultimately to improve the success of novel therapies in combatting AMR.

## Pasteur's legacy

In this Editorial, I have celebrated Pasteur's impact on infectious disease, highlighting a key role for ‘l'esprit Pasteur’ in preparing for and fighting ongoing and future pandemics. The availability of innovative infection platforms and animal models has transformed approaches to study human disease, and their full potential has yet to be realised (López-Jiménez and Mostowy, 2021; Tobin, 2022). It is important for the modern scientific community to discuss how researchers using laboratory infection models can position themselves for maximum impact, and how they can empower science and society for future emerging threats. A more complete understanding of microbiology, in combination with innovative technologies and collaboration with other disciplines, such as systems biology and human immunology, will inevitably transform our pandemic preparedness. By openly communicating this research and fostering collaboration, journals such as DMM can be viewed as important stakeholders in the space of infectious disease and can help this research to impact human health. This can be achieved through open-access publication and by sponsoring and organising conferences, journal meetings and workshops, such as DMM's 2023 meeting, ‘Infectious Diseases Through an Evolutionary Lens’, in London.

In celebration of the bicentenary of Pasteur's birth, it is exciting to consider his legacy in shaping the future of microbiology. There are many outstanding questions in infectious disease, inspired by Pasteur. What is the role of chemistry in future microbiology? What technological innovations can help to revolutionise future microbiology? How can investigation of exotic bacterial species promote our pandemic preparedness? How will advances in the field shape future medicine? As our fundamental and biomedical knowledge continues to improve, the future of microbiology should be motivated to enable transformative discovery with translational applications.
